# Supplementary figures and images for: Global, regional and national burden of injuries caused by fire, heat, and hot substances from 1990 to 2021
Source: PLoS One. 2025 May 20;20(5):e0324481. doi: 10.1371/journal.pone.0324481 (PMC12091820; doi:10.1371/journal.pone.0324481)

A.

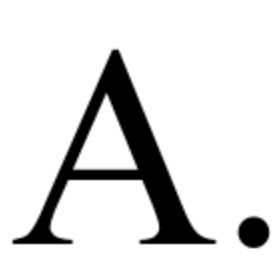

B.

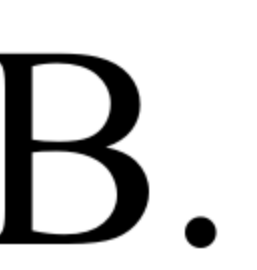

C.

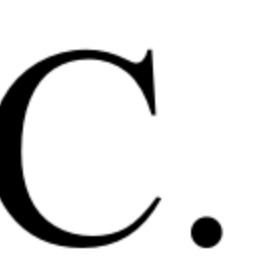

D.

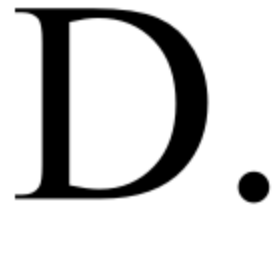

## E.

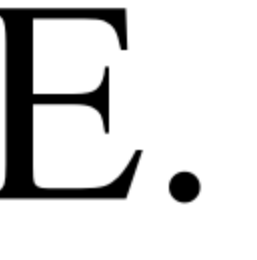

F.

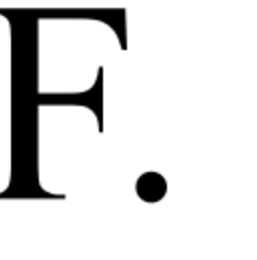

Supplement: S1 Fig — A. is for the Age-Standardized Rate of Incidence; B. is for the Age-Standardized Rate of Prevalence; C. is for the Age-Standardized Rate of Death; D. is for the Age-Standardized Rate of Disability-Adjusted Life Years. (E) The Age-Standardized Rate of Years of Life Lost; F. is for the Age-Standardized Rate of Years Lived with Disability. (PDF) [file pone.0324481.s001.pdf]
