# Supplementary material for: Global, regional and national burden of injuries caused by fire, heat, and hot substances from 1990 to 2021
Source: PLoS One. 2025 May 20;20(5):e0324481. doi: 10.1371/journal.pone.0324481 (PMC12091820; doi:10.1371/journal.pone.0324481)
Supplement: S1 File — Formulas of Age-Standardized Rate, Estimated Annual Percentage Change, Frontier Analysis, and Bayesian Age - Period - Cohort model. (DOCX) [file pone.0324481.s002.docx]

# Chapter1: Calculation of ASR and EAPC^1,2^

*ASR =* $\frac{\sum(X_{age}\times W_{age})}{\sum W_{age}}$

*"X_age_"* refers to the Number of Incidences, Deaths, Prevalence, DALYs, YLLs, and YLDs (where “Number” here refers to the count of individuals or years). *“Wage”* refers to the proportion of the corresponding age group in the standard population.

*EAPC =*$100\times[exp(\beta)-1]$

*β* is the regression coefficient obtained by performing a linear regression analysis on the logarithmically transformed age-standardized rates *[Ln (ASR)]* corresponding to each year (time)^3^ . The specific steps are as follows:

*Ln (ASR) =* $\alpha+\beta\times y$*ear+*$\sigma$

*α* is the intercept, *β* is the slope (i.e., the regression coefficient), and *σ* is the error term.

# Chapter2: Frontier Analysis^3–5^

Frontier analysis is a crucial quantitative tool in burden of disease research for analyzing the relationship between disease burden and population development. In this study, we assess and optimize health system efficiency using Data Envelopment Analysis (DEA) and Locally Estimated Scatterplot Smoothing (LOESS) techniques. Specifically, we use the Free Disposal Hull (FDH) method for DEA to plot a non-linear frontier.

1. We begin by identifying the lowest possible burn burden through data processing, extracting burn data from the Global Burden of Disease database and calculating the average burn DALYs rate for each SDI value using 1,000 bootstrapped samples.

2. We then smooth the frontier boundary using LOESS with a polynomial degree of 1 and a span of 0.2, excluding super-efficient countries to avoid the impact of outliers.

3. Effective Difference, which measures the absolute distance to the frontier, is used to quantify efficiency differences; for countries or regions with ASR values below the frontier, this difference is set to zero.

4. A larger Effective Difference indicates a greater room for improvement in burn control, suggesting that these countries or regions can reduce the burden of burns by optimizing policy and resource allocation.

# Chapter3: BAPC Model^6,7^

Within the Bayesian Age-Period-Cohort (BAPC) model, the interplay of its constituent elements forms a comprehensive analytical framework. At its core lies the log-linear Poisson model, which delineates the variation in health outcomes, such as incidence rates or mortality rates, as a function of age ($\alpha_{i}$), period ($\beta_{j}$), and cohort ($\gamma_{k}$). This model posits that health outcomes follow a log-linear Poisson distribution, with $\epsilon_{ijk}$ representing the unexplained stochastic variation, typically assumed to be normally distributed.

Subsequently, the prior distribution $p\left( \theta\right)$ in Bayesian statistics encapsulates our pre-data beliefs about the parameters, informed by historical data, ancillary information, or the expertise and knowledge of researchers. The prior distributions for age, period, and cohort effects are often specified as inverse gamma distributions. Following this, the posterior distribution $p\left( \theta| y \right)$ is derived by integrating observational data with prior knowledge through Bayes' theorem, reflecting our updated beliefs about the parameters in light of the observed data. This distribution signifies the influence of the data on our understanding of the parameters.

Moreover, to address overdispersion, the BAPC model may employ a second-order random walk (RW2) model as a prior distribution, adjusting for period effects based on the assumption of a linear temporal trend. This prior distribution aids in smoothing period effects and mitigating overdispersion in the model.

Lastly, the Integrated Nested Laplace Approximation (INLA) is a computational method used to approximate the posterior distributions of Bayesian models, particularly suited for high-dimensional and complex spatio-temporal models like the BAPC model. INLA offers an efficient approach to handling a multitude of parameters within the model and provides rapid and accurate estimates of the posterior distributions. Collectively, these components enable the BAPC model to analyze and forecast the shifting burdens of disease in relation to age, period, and cohort, offering a holistic and adaptable methodology for investigating intricate epidemiological patterns.

$\log{(\lambda}_{ijk})=$ $\mu+\alpha_{i}+\beta_{j}+\gamma_{k}+\epsilon_{ijk}$

In the formula, $\lambda_{\mathrm{ijk}}$ represents the expected value of the burn burden for the i-th age group, the j-th period, and the k-th cohort; $\mu$ is the constant term, indicating the baseline level of the logarithm of incidence or mortality rate when all other effects are assumed to be zero; $\alpha_{i}$ is the age effect, which signifies the impact of age on the burden of burn injury; $\beta_{j}$ is the period effect, which signifies the impact of the period on the burden of burn injury; $\gamma_{k}$ is the cohort effect, , which signifies the impact of the birth cohort on the burden of burn; $\epsilon_{\mathrm{ijk}}$ is the random error, which represents the random variation that the model fails to explain and is typically assumed to be normally distributed.

$$F\left( \chi;\alpha,\beta\right)=\frac{\beta^{\alpha}}{r\left( \alpha\right)}\chi^{-\alpha-1}{e^{-}}^{\frac{\beta}{\chi}}$$

$\chi$ is a random variable following the inverse gamma distribution, which is commonly used to model the variance of a normal distribution; α represents the shape parameter of the inverse gamma distribution; β denotes the scale parameter of the inverse gamma distribution; and $r\left( \alpha\right)$ refers to the gamma function, a generalization of the factorial function to real and complex numbers.

$$p\left( \theta| y \right) \alpha p\left( \theta| y \right)p\left( \theta\right)$$

The posterior distribution of the parameter θ given the data y, where p(y|θ) is the likelihood function, representing the probability of observing the data y under the given parameter θ, and p(θ) is the prior distribution of the parameter θ, representing the belief about the parameter before observing the data.

$$\beta_{j}=\beta_{j-1}+\eta_{j}$$

βj represents the effect of the j-th period. βj−1 denotes the effect of the previous period, which is the (j-1)-th period. ηj is the random variation from the (j-1)-th period to the j-th period, which is typically assumed to be normally distributed.

# Reference

1. Wyper GMA, Grant I, Fletcher E, McCartney G, Fischbacher C, Stockton DL. How do world and European standard populations impact burden of disease studies? A case study of disability-adjusted life years (DALYs) in Scotland. *Archives of Public Health*. 2020;78:1.

2. WHO methods and data sources for global burden of disease estimates 2000-2019. Global Health Estimates Technical Paper WHO/ DDI/DNA/GHE/2020.3.

3. Yu Y, Li H, Hu N xi, et al. Global burden and health inequality of nutritional deficiencies from 1990 to 2019. *Front Nutr*. 2024;11.

4. Chen X, Zhou CW, Fu YY, et al. Global, regional, and national burden of chronic respiratory diseases and associated risk factors, 1990-2019: Results from the Global Burden of Disease Study 2019. *Front Med (Lausanne)*. 2023;10:1066804.

5. Luh J, Cronk R, Bartram J. Assessing Progress towards Public Health, Human Rights, and International Development Goals Using Frontier Analysis. *PLOS ONE*. 2016;11(1):e0147663.

6. Riebler A, Held L. Projecting the future burden of cancer: Bayesian age–period–cohort analysis with integrated nested Laplace approximations. *Biometrical J*. 2017;59(3):531-549.

7. Fosse E. Bayesian age–period–cohort models. In: Bell A, ed. *Age, Period and Cohort Effects*. 1st ed. Routledge; 2020:142-175.
